# Development and validation of a liquid chromatography coupled to a diode array detector (LC-DAD) method for measuring mitotane (DDD) in plasma samples

**DOI:** 10.1016/j.clinsp.2024.100470

**Published:** 2024-08-10

**Authors:** Anna Sylvia Ferrari Marques, Atecla Nunciata Lopes Alves, Berenice Bilharinho Mendonca, Helena Panteliou Lima-Valassi

**Affiliations:** a Laboratório de Hormônios e Genética Molecular LIM-42, Hospital das Clínicas da Faculdade de Medicina da Universidade de São Paulo (HCFMUSP), São Paulo, SP, Brazil; b Núcleo Multiusuário de Cromatografia Líquida Associada à Espectrometria de Massas em Tandem, (AE-06 Rede Premium), Brazil

**Keywords:** Adrenocortical carcinoma, Mitotane, Liquid chromatography, Diode array, Analytical, Method validation, HPLC

## Abstract

**Introduction:**

Mitotane (o,p’–DDD) is the drug of choice for Adrenocortical Carcinomas (ACC) and its measurement in plasma is essential to control drug administration.

**Objective:**

To develop and validate a simple, reliable and straightforward method for mitotane determination in plasma samples.

**Method:**

Drug-free plasma samples were collected in potassium-ethylenediamine tetraacetate (K-EDTA) tubes and spiked with 1.0, 2.5, 10.0, 25.0 and 50.0 µg/mL of mitotane (DDD). The p,p’-DDD was used as an Internal Standard (IS) and was added at 25.0 µg/mL concentration to all samples, standards and controls. Samples were submitted to protein precipitation with acetonitrile and then centrifuged. 50 uL of the supernatant was injected into an HPLC system coupled to a Diode Array Detector (DAD). DDD and IS were detected at 230 nm in a 12 min isocratic mode with a solvent mixture of 60 % acetonitrile and 40 % formic acid in water with 0.1 % pump mixed, at 0.6 mL/min flow rate, in a reversed-phase (C18) chromatographic column kept at 28°C. The sensitivity, selectivity, precision, presence of carry-over, recovery and matrix-effect, linearity, and method accuracy were evaluated.

**Results:**

The present study's method resulted in a symmetrical peak shape and good baseline resolution for DDD (mitotane) and 4,4’-DDD (internal standard) with retention times of 6.0 min, 6.4 mim, respectively, with resolutions higher than 1.0. Endogenous plasma compounds did not interfere with the evaluated peaks when blank plasma and spiked plasma with standards were compared. Linearity was assessed over the range of 1.00–50.00 µg/mL for mitotane (R2 > 0.9987 and a 97.80 %‒105.50 % of extraction efficiency). Analytical sensitivity was 0.98 µg/mL. Functional sensitivity (LOQ) was 1.00 µg/L, intra-assay and inter-assay coefficient of variations were less than 9.98 %, and carry-over was not observed for this method. Recovery ranged from 98.00 % to 117.00 %, linearity ranged from 95.00 % to 119.00 %, and high accuracy of 89.40 % to 105.90 % with no matrix effects or interference was observed for mitotane measurements. Patients' sample results were compared with previous measurements by the GC-MS method with a high correlation (*r* = 0.88 and bias = -10.20 %).

**Conclusion:**

DDD determination in plasma samples by the developed and validated method is simple, robust, efficient, and sensitive for therapeutic drug monitoring and dose management to achieve a therapeutic index of mitotane in patients with adrenocortical cancer.

## Highlights

• A simple and sensitive HPLC-DAD has been developed and validated for mitotone determination in plasma.
• Plasma concentration of mitotane was assayed in adrenocortical cancer patients.
• The assay is suitable for the therapeutic drug monitoring of mitotane in clinical settings.

## Introduction

**Fig. 1:**
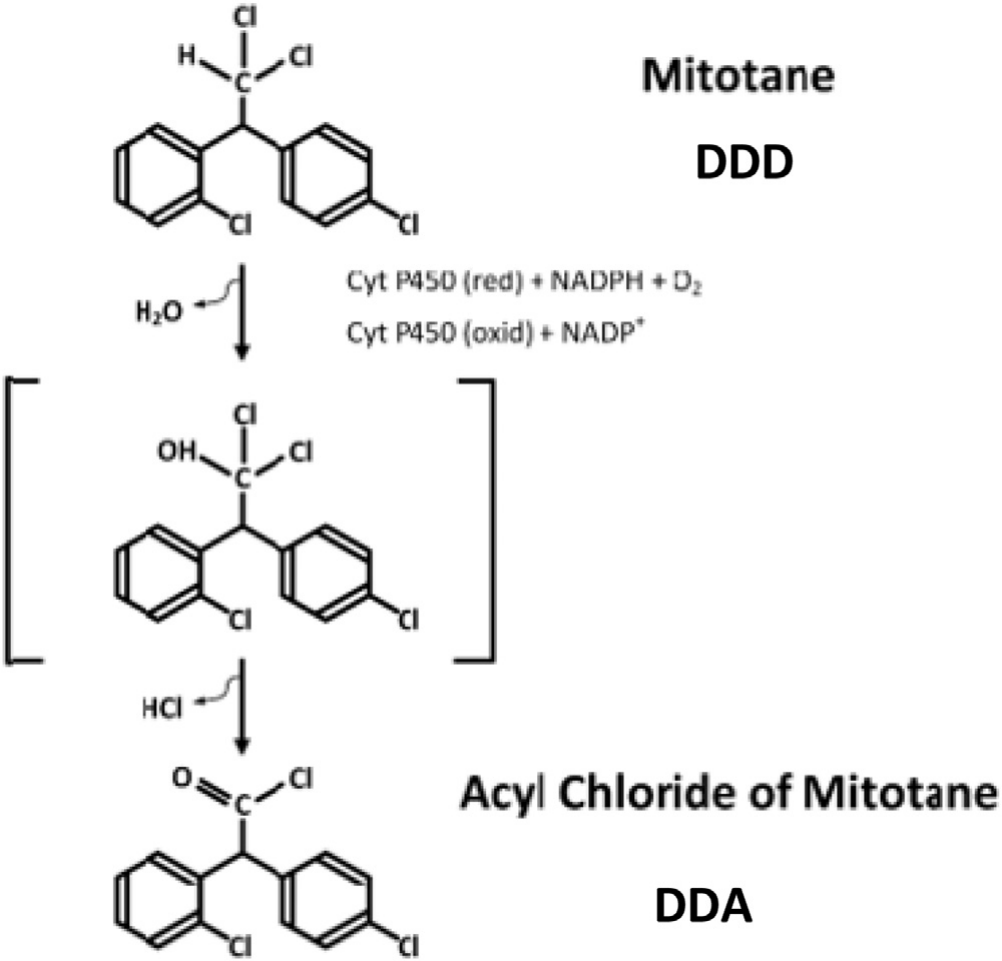
Mitotane metabolism by means of cytochrome P450 enzyme.

**Fig. 2:**
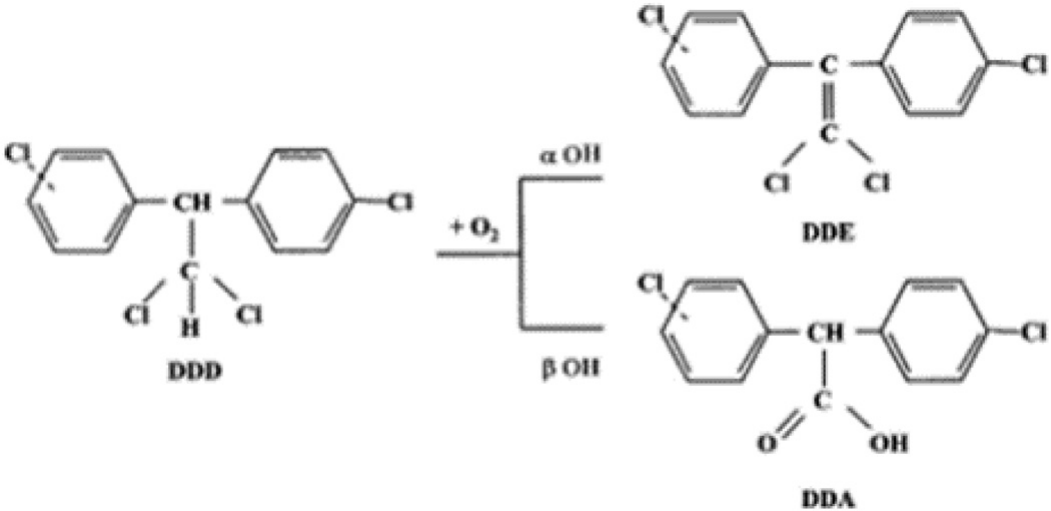
Mitotane and its metabolites DDA and DDE.

Adrenocortical Carcinoma (ACC) is a rare endocrine malignancy arising from one of the three cortical layers of the adrenal gland. ACCs could cause an increase in the production of one or more steroid hormones, such as cortisol, androgens, and aldosterone, resulting in clinical manifestations of Cushing's syndrome, virilization, and high blood pressure.^1^ Mitotane (o,p’–DDD) or (1,1-dichloro-2-[o-chlorophenyl]-2-[p-chlorophenyl]ethane, DDD) is the only approved drug for Adrenocortical Carcinomas (ACC) treatment. Intra-adrenal metabolic transformation is essential for the therapeutic effect of DDD, which is metabolized to DDE (o,p’-DDE) and DDA (o,p’-DDA) by α- and β-hydroxylation, respectively (Fig. 1, Fig. 2). Mitotane is metabolized through a reactive acyl chloride thought to bind adrenal cortical bionucleophiles as well as to serve as the intermediate in the formation of o,p’-DDA, probably by means of a Cytochrome P450 (CYP) enzyme.^2^, ^3^, ^4^

The exact DDD mechanism of action is still under research as well as the complete role of metabolites related to DDD: DDA and DDE.^5^, ^6^, ^7^ Mitotane has a narrow therapeutic index, plasma level monitoring of mitotane during treatment allows the more rapid attainment of a therapeutic plasma level (14–20 µg/mL), as well as minimization of adverse effects due to an excessive high plasma level.^6^ Mitotane levels of more than 20 mg/L are associated, also in children, with neurological toxicity.^8^^,^^9^ Currently, mitotane is the only adrenolytic drug, as a single or adjuvant treatment, for severe and advanced ACC.^10^

Due to the high lipophilic nature of DDD, sample preparation and liquid chromatographic analysis becomes quite challenging.^11^ In the pool of hydrophobic drugs, mitotane also suffers from intrinsic poor aqueous solubility and low bioavailability.^12^ HPLC methods constitute a valid alternative to gas chromatography^13^ in monitoring plasma DDD levels with less laborious sample preparation and faster method acquisition. The objective of this work was to develop and validate a simple, reliable, and straightforward method for mitotane determination in plasma samples.

## Materials and methods

### Chemicals and reagents

DDD and 4,4’-DDD were purchased from Chem Service Inc. (USA). Methanol, formic acid and acetonitrile, HPLC grade solvents, were purchased from Supelco (Merck KGaA, Darmastadt, Germany). Water was obtained by a MilliQ Integral 15, A10 TOC Monitor, (Millipore, Merck KGaA, Darmastadt, Germany). All stock standard solutions of compounds were prepared in methanol at a concentration of 1 mg/mL and stored at -20 °C.

### Samples

Drug-free plasma samples collected in potassium-ethylenediamine tetraacetate (K-EDTA) tubes were spiked with 1.0, 2.5. 10.0, 25.0 and 50.0 µg/mL levels of DDD. A pool of patient samples at low and high concentrations was selected to provide two levels of control. Two hundred µL of plasma samples, standards and controls were transferred into microcentrifuge tubes. Proteins were precipitated by the addition of 2× volumes (400 µL) of acetonitrile containing 25 µg/mL of 4,4’-DDD for Internal Standard (IS) and vortexed for 20 s. The mixture was centrifuged, for a second time, at 14500 × g for 15 min in a Herolab MicroCen16 microcentrifuge (GmbH Laborgeräte, Germany). To the supernatant 100 µL of 0.1 % formic acid in water was added, vortex mixed and centrifuged at 14500 × g for 15 min again. The supernatant was transferred to glass autosampler vials and 50 µL was injected into the chromatography system.

### Chromatographic system and conditions

Chromatography was carried out in an Agilent Infinity 1260 II system equipped with a 1260 Flexible Pump quaternary piston pump, 1260 MCT column oven, 1260 Multisampler autoinjector, and a DAD HS variable wavelength UV detector (Agilent Technologies, Santa Clara, CA, USA). Data acquisition and integration were performed using OpenLab software computer-based integration system to determine peak areas. Separation achieved by an isocratic mode with a solvent mixture of A: 60 % acetonitrile and B: 40 % formic acid in water 0.1 %, pump mixed, at 0.6 mL/min flow rate through a Waters Acquity HSS T3 (100 × 2.1 mm, 5 µm) (Waters Technologies, Milford, MA, USA) and kept at 28°C as well as a pre-column Phenomenex SecurityGuard ULTRA C18 (Phenomenex, Torrance, CA, USA). The total analysis time was 12 min and DAD detection at 230 nm.

### Method validation

Was performed according to guidelines for bioanalytical method validation by Clinical Laboratories Standards Institute (CLSI) guidelines^14^ and RDC 27/2012, RDC 166/2017 – ANVISA^15^^,^^16^ and included: analytical and functional sensitivity, selectivity, linearity, precision, accuracy, recovery, matrix effect, interference assessment and carryover, which are described below.

Analytical sensitivity was defined as the lowest concentration which is different from a blank sample. Functional sensitivity was defined as the lowest concentration with inter-assay variation of less than 20 %. Linearity was assessed by mixing in different proportions a high mitotane concentration sample with a low mitotane concentration sample. Precision studies were determined with two different concentration samples, for intra-assay 10 aliquots of each sample were analyzed in the same day, and for inter-assay 20 replicates were analyzed over five days. Accuracy was estimated by recovery measurement adding standards to three samples without DDD and compared with reference method (GCMS) and expected results. Recovery and matrix effect was assessed by comparison between the slopes obtained in solvent and in real matrix (standard additions). Samples containing DDA and DDE, metabolites of DDD, were tested in this method for assessment of potential interference. Carryover was evaluated when the potential preceding elevated concentration of the analyte could interfere with the results. It was investigated by assaying two analyte specimens with low and high concentrations and comparing SD (Standard Deviation) of 11 replicates with low concentration and 10 replicates with high concentration in the following order: 3 low, 2 high, 1 low, 2 high, 4 low, 2 high, 1 low, 2 high, 1 low, 2 high and 1 low sample. DDD stability was evaluated in plasma exposed to different conditions: 0, 2, 3, 5 and 7 days at room temperature, 10 °C and -20 °C. Results were analyzed using EP evaluator (David G. Rhoads Associates, Inc, Kennett Square, PA) or EXCEL® software (Microsoft Corporation, USA).

## Results and discussion

**Fig. 3:**
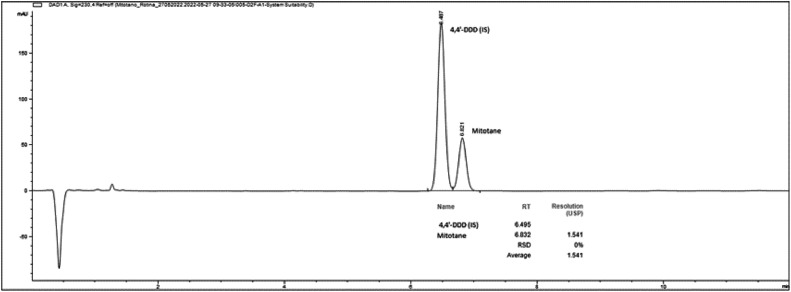
Chromatogram obtained showing a symmetrical peak shape and good baseline resolution for DDD (mitotane) and 4,4’-DDD (internal standard) with retention times of 6.0 min, 6.4 mim, respectively.

Due to the high hydrophobicity, and non-polar chemical nature of mitotane (DDD) the reverse-phase chromatography mode was the best choice. Different octadecyl silica (C18) column chemistries were also assessed and the Waters HSS T3 C18, 1.7 µm particle size showed the best resolution between DDD and internal standard 4,4’-DDD under an isocratic mode as opposed to a gradient elution mode. It was considered the high chemical similarities between DDD and 4,4’-DDD molecule structures in order to choose the best internal standard and under optimal developed conditions it showed appropriate resolution. As for mobile phase selection, acetonitrile was chosen as the organic modifier and the mobile phase pH was kept at 2.3 with 0.1 % formic acid in water leading to very good peak shapes at a flow rate of 0.6 mL/min. Symmetrical peak shape and good baseline resolution for DDD and 4,4’-DDD were obtained with retention times of 6.0 min and 6.4 mim, respectively, with a peak resolution higher than 1.0 (Fig. 3). Final mobile phase combination was 40 % of 0.1 % formic acid in water and 60 % acetonitrile (v/v), pump mixed. The authors found that both DDD and 4,4’-DDD gave better UV absorbance at 230 nm wavelength, compared to 226 nm wavelength. The column temperature was investigated and set at a nominal value of 28 °C, an essential parameter for small particle UPLC columns avoiding chromatographic band spread promoted by mobile phase/particle attrite. As for the sample preparation, acetonitrile was the best solvent to promote protein precipitation and high DDD recoveries, with a final addition of acidified water phase, 0.1 % formic acid solution. Recovery values confirmed good method accuracy with no matrix effect (> 0.99 correlation) as well as method selectivity.

**Fig. 4:**
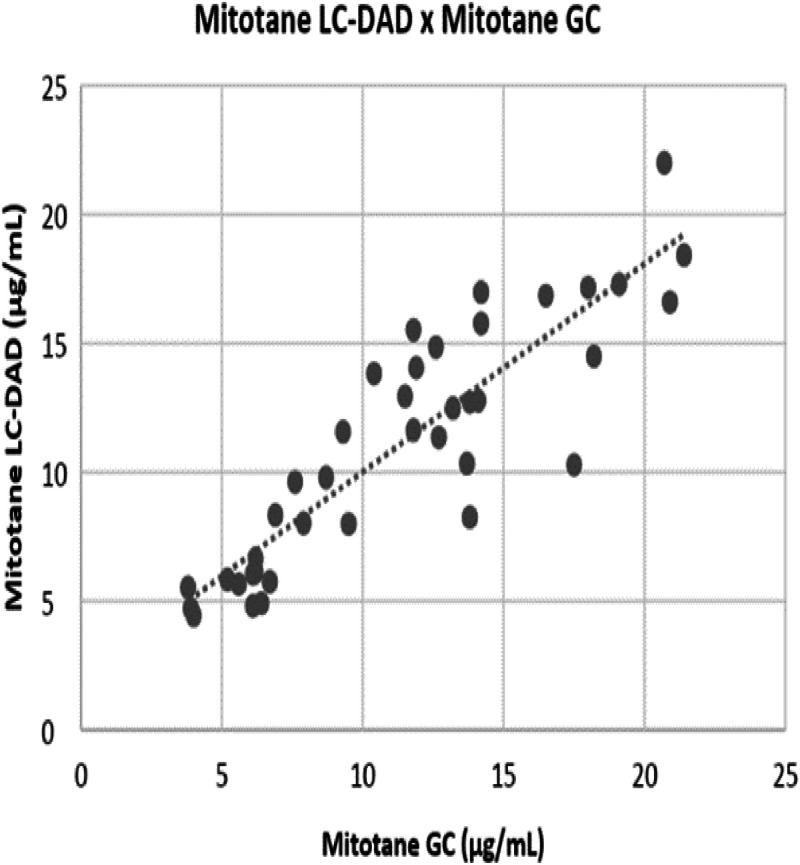
Correlation between results obtained by LC-DAD and GC-MS (gold standard). (*r* = 0.88 and bias = -10.2)

The standard curve was linear over the studied concentration range (1.0 to 50.0 µg/mL) with R^2^ > 0.9987 and a 97.8 % to 105.5 % extraction efficiency. Analytical sensitivity (limit of quantification, LOD) was 0.976 µg/mL and functional sensitivity (lower limit of quantitation, LOQ) was defined as approximately 1.00 µg/mL. DDD metabolites did not interfere with this method. Precision was expressed 3.89 %, 2.28 %, and 2.43 % for intra-assay evaluations and 9.98 %, 5.39 % and 5.01 % for inter-assay. Accuracy ranged from 89.4–105.9 % (Table 1). Patient samples were previously assessed at a supporting laboratory collaboration with a 0.88 results correlation and -10.2 bias against a GC-MS method (Fig. 4).

**Table 1:** Characteristics of the LC-DAD method and acceptance criteria.

Parameter	Result	Acceptance criteria
Analytical sensitivity	0.98 µg/mL	‒
Functional sensitivity	1.00 µg/mL	‒
Intra-assay precision	2.3 % to 3.9 %	15 %
Inter-assay precision	3.6 % to 6.4 %	15 %
Carryover	Not detected	No carry over
Recovery	98 % to 117 %	80 %‒120 %
Accuracy	89 % to 106 %	80 %‒120 %
Linearity	98 % to 106 % (R^2^ > 0.9987)	80 %‒120 %

Mitotane was also evaluated for stability testing as part of the validation studies. Five patient samples were selected for the testing. The sets were stored in Room Temperature (Day 0‒7), Fridge (Day 0‒15), and Freeze-Thaw (Day 0‒30). The results for room temperature showed the highest variation throughout the days. From Day 0‒2, showed variations as low as 0.90 % and as high as 27.12 % past 7 days of exposure. As for Fridge exposure, from Day 0‒2 showed variations as low as 1.68 % and as high as 19.05 % for Day 15. For Freeze-Thaw exposure, the variations for Days 0‒30 were as high as 16.97 %, past the 30 days. All the variations were within the 20 % acceptance criteria, except for room temperature, which after 7 days sample showed degradation.

## Conclusion

LC-DAD developed, and validated method is a simple, robust, efficient, and sensitive method to measure mitotane levels in patient plasma samples and it can be an alternative to GC-MS methods with less laborious sample preparation and shorter run time. The method has been evaluated using samples from more than 35 adrenocortical cancer patients on mitotane therapy. The method proved to be consistent, and it is ideally suited for therapeutic drug monitoring enabling dose modification to achieve mitotane therapeutic index.

## Ethical approval

This study was approved by the Medical Ethical Committee of the Clinical Direction Board from Hospital das Clinicas, Medicine Faculty São Paulo University (2023). All procedures performed in this study were in accordance with the ethical standards of the institutional medical ethics committee.

## Institutional review board statement

The study required ethical approval by Plataforma Brasil, CEP CAAE n° 62465721.0.0000.0068, document registration number: 6.053.869.

## Informed consent statement

The study did not require an Informed Consent Statement.

## Declaration of competing interest

The authors declare no conflicts of interest.
